# Compliance monitoring in business processes: Functionalities, application, and tool-support

**DOI:** 10.1016/j.is.2015.02.007

**Published:** 2015-12

**Authors:** Linh Thao Ly, Fabrizio Maria Maggi, Marco Montali, Stefanie Rinderle-Ma, Wil M.P. van der Aalst

**Affiliations:** a Ulm University, Germany; b University of Tartu, Estonia; c Free University of Bozen-Bolzano, Italy; d University of Vienna, Faculty of Computer Science, Austria; e Eindhoven University of Technology, The Netherlands

**Keywords:** Business process compliance, Compliance monitoring, Operational support

## Abstract

In recent years, monitoring the compliance of business processes with relevant regulations, constraints, and rules during runtime has evolved as major concern in literature and practice. Monitoring not only refers to continuously observing possible compliance violations, but also includes the ability to provide fine-grained feedback and to predict possible compliance violations in the future. The body of literature on business process compliance is large and approaches specifically addressing process monitoring are hard to identify. Moreover, proper means for the systematic comparison of these approaches are missing. Hence, it is unclear which approaches are suitable for particular scenarios. The goal of this paper is to define a framework for Compliance Monitoring Functionalities (CMF) that enables the systematic comparison of existing and new approaches for monitoring compliance rules over business processes during runtime. To define the scope of the framework, at first, related areas are identified and discussed. The CMFs are harvested based on a systematic literature review and five selected case studies. The appropriateness of the selection of CMFs is demonstrated in two ways: (a) a systematic comparison with pattern-based compliance approaches and (b) a classification of existing compliance monitoring approaches using the CMFs. Moreover, the application of the CMFs is showcased using three existing tools that are applied to two realistic data sets. Overall, the CMF framework provides powerful means to position existing and future compliance monitoring approaches.

## Highlights

• Framework of ten compliance monitoring functionalities (CMF) in business processes is defined.
• Related techniques and enabling technologies are discussed.
• Systematic literature review and presentation of real-world business constraints for compliance are extracted from five case studies.
• Systematic comparison with compliance patterns and classification of existing approaches using the framework is presented.
• CMF framework based on two realistic data sets using three different compliance monitoring tools is applied.

## 1 Introduction

Business process compliance emerged as hot topic in research during the last few years. In essence, several approaches have been developed to formally and (semi-) automatically prove that business processes comply with relevant constraints such as regulations, laws, or guidelines. An example constraint from the medical domain would be “The patient has to be informed about the risks of a surgery before the surgery takes place”. In practice, compliance checks are often conducted manually and hence perceived as a burden [1], although their importance is undoubted.

The need to check for compliance of business processes based on a set of constraints may emerge in different phases of the process life cycle [2,3]. During design time, the compliance of a process model with a set of constraints is checked. At runtime, the progress of a potentially large number of process instances is monitored to detect or even predict compliance violations. For this, typically, terms such as compliance monitoring or online auditing are used. Finally, processes can be diagnosed for compliance violations in a *post mortem* or offline manner, i.e., after process instance execution has been finished.

**Fig. 1:**
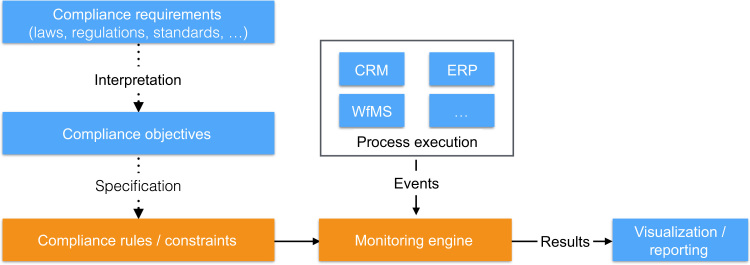
Compliance monitoring for business processes: general approach.

This paper is dedicated to compliance monitoring as this is crucial for the timely detection and prediction of compliance violations as well as for the provision of reactive and pro-active countermeasures on compliance violations [4–6]. Further, in realistic settings, the existence of a complete process model for compliance checks cannot always be assumed. In fact, business processes are often implemented in a rather implicit manner and executed over different information systems (e.g., Enterprise Resource Planning (ERP) or Customer Relationship Management (CRM) tools) as depicted in Fig. 1. Although there are similarities between design time/*post mortem* analysis and compliance monitoring (see also Section 3.1.5), this paper will focus on the latter in order to provide a clear scope.

Typically, compliance requirements on business processes stem from different sources such as laws, regulations, or guidelines that are often available as textual descriptions. An important task towards compliance monitoring is the interpretation of these requirements as compliance objectives and the subsequent specification as compliance rules or constraints (note that, in this paper, we will use both terms interchangeably). As shown in Fig. 1, the specified compliance rules will be verified over the process execution events. The results of compliance monitoring can be visualized and reported back to users in different ways, ranging from notifications on violations to fine-grained feedback on reasons for violations, or even the prediction of possible and unavoidable future violations.

In general, compliance monitoring approaches are driven by two factors: (1) the *compliance rule language* that is used to specify the compliance requirements and (2) the *event format* the compliance checks are based on. Due to the possible heterogeneity of the data sources employed, an integrated target event format is desirable.

### 1.1 Problem statement

There is a overwhelming body of literature on business process compliance. The approaches address different phases of the process life cycle and often propose different languages. These are used to formally represent the constraints to be checked on the business processes. Overall, it is hard to oversee and compare the already existing approaches and hence, the decision of which approach could be utilized for which kind of problem is hampered.

Hence, the main challenge tackled in this paper is to provide proper means for comparing approaches for compliance monitoring in business processes in a systematic way. This challenge will be addressed by the following four research questions. The first one refers to the challenge of identifying approaches for compliance monitoring and to distinguish them from approaches that provide design time compliance checks, *post mortem* conformance checking, compliance checking architectures, or mention compliance monitoring as an important building block

(↦ *Research Question* 1 (*RQ*1): *How to identify compliance monitoring approaches?*).

This is important to provide the study with a clear focus.

The second research question fosters the derivation of a set of typical functionalities required in compliance monitoring approaches and practice

(↦ *Research Question* 2 (RQ2): *What are functionalities that are essential for compliance monitoring approaches in business processes?*).

RQ2 meets the challenge of granularity and coverage.

The challenge of how to demonstrate the appropriateness of the identified functionalities is picked up by the third research question

(↦ *Research Question* 3 (*RQ3*): *How can we demonstrate the appropriateness of the identified compliance monitoring functionalities*?).

The fourth research question asks for the applicability of the functionalities

(↦ *Research Question* 4 (*RQ4*): *How can the compliance monitoring functionalities be applied in existing tools*?).

Section 1.2 discusses the applied methodology and gives an overview on how RQ1 to RQ4 will be tackled in this paper.

### 1.2 Research methodology

**Fig. 2:**
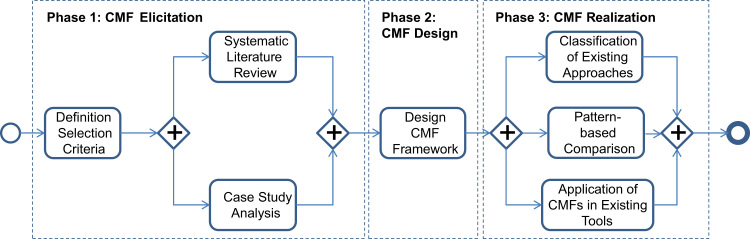
Methodology for the elicitation, design, and realization of CMFs (in BPMN notation).

The goal of this paper is to define a framework for Compliance Monitoring Functionalities (CMF) that enables the systematic comparison of existing and new approaches for monitoring compliance rules over business processes during runtime. Specific challenges for eliciting the CMFs are the multitude of existing approaches in the area of business process compliance and the decision of which functionalities are required in real-world scenarios. In order to address these challenges, we apply the methodology depicted in Fig. 2.

The methodology consists of three phases, i.e., *elicitation*, *design*, and *realization* of the CMF framework.

*Phase 1—Elicitation*: The elicitation phase follows the research methodology described in the context of elicitation of process change and time patterns [13,14]. First of all, *selection criteria* are defined that scope the research done in this paper (↦ RQ1 and RQ2). As overarching selection criteria, we focus on:

1. *functionalities that are relevant for process compliance monitoring*, i.e., the observation and enforcement of compliance constraints that are imposed over business processes during *runtime* and
2. *constraints that are imposed at the process level*, i.e., we exclude, for example, integrity constraints [15].

The elicitation phase includes a systematic literature review described in Section 3.1 and an analysis of five case studies from different domains introduced in Section 3.2. The CMF identification is based on the results of the systematic literature review and the case study analysis (↦ RQ2) and possibly illustrated by additional examples.

*Phase* 2—*Design*: The CMF design itself is presented in Section 4. Each CMF is described using a CMF template and illustrated by examples taken from literature or case studies (↦ RQ2).

*Phase* 3—*Realization*: In order to demonstrate the appropriateness of the CMF design, existing approaches are classified along their support for the CMFs (cf. Section 4). Moreover, constraint patterns as suggested by the literature are compared to the CMFs proposed in this paper (↦ RQ3). Finally, to illustrate the application of the CMF framework, compliance rules are extracted from two realistic and publicly available data sets. Based on the extracted rules and the data sets we showcase the application of compliance monitoring for business processes in existing tools (cf. Section 5.3) (↦ RQ4). Note that the data sets for CMF application are different from the case studies utilized for CMF elicitation.

### 1.3 Contribution

In this paper, we address research questions RQ1 to RQ4 as stated Section 1.1. The core of the approach is a framework of functionalities that are relevant in the context of compliance monitoring. These *Compliance Monitoring Functionalities* are denoted as *CMFs* for short. The *CMF Framework* (*CMFF* for short) shall enable a systematic comparison of existing as well as new approaches on compliance monitoring in business processes. In summary, the main contributions of this paper are

• A systematic literature review on compliance monitoring approaches in business processes and analysis of selected projects from different domains (↦ RQ1 and RQ2).^⁎⁎^
• A framework (CMFF) based on 10 compliance monitoring functionalities described in a systematic way (↦ RQ2).^⁎^
• A comparative survey of typical compliance rule patterns found in the literature highlighting the importance of the selected CMFs (↦ RQ3).
• A detailed analysis and discussion of compliance monitoring approaches based on the CMFF (↦ RQ3).^⁎⁎^
• The application of the CMFF using existing tools and two realistic data sets (↦ RQ4).

The ^⁎^ and ^⁎⁎^ annotations of selected contributions highlight the extensions made on the previous EDOC 2013 conference paper [10]. Here, ^⁎^ means that the contribution is based on [10] and ^⁎⁎^ means an extension of the particular contribution when compared to [10]. Contributions without annotations are entirely new.

The remainder of the paper is structured as follows. In Section 2, a short introduction to compliance monitoring in the context of process mining is provided. Section 3 describes the research methodology followed and discusses approaches that are closely related to compliance monitoring. Section 4 describes our CMFF composed of ten CMFs. In Section 5, we focus approaches to concretely support CMFs. First of all, a comparison of the CMFF with pattern-based approaches is presented. Furthermore, existing compliance monitoring approaches are classified using the CMFs. Finally, the application of the CMFF based on two realistic data sets within three selected tools is showcased. Section 6 concludes the paper.

## 2 Compliance monitoring in the context of process mining

The basic idea behind process mining is to discover, monitor and improve processes by extracting knowledge from data that is available in today׳s systems [99]. The starting point for process mining is an *event log*. XES (eXtensible Event Stream) [7,8] has been developed as the standard for storing, exchanging and analyzing event logs. Each event in a log refers to an activity (i.e., a well-defined step in some process) and is related to a particular case (i.e., a process instance). The events belonging to a case are ordered. Hence, a case can be viewed as a sequence of events (i.e., a trace). Event logs may store additional information about events such as the resource (i.e., person or device) executing or initiating the activity, the timestamp of the event, or data elements recorded with the event.

The reference framework presented in [99] gives an overview of the process mining spectrum. The event logs are partitioned into two kinds: *pre mortem* and *post mortem. Pre mortem* logs refer to current process instances that are ongoing; *post mortem logs* refer to historical process instances that have completed. The framework also distinguishes two types of models: *de jure* and *de facto*. A *de jure* model is normative, i.e., it specifies how things should be done or handled. A *de facto* model is descriptive and its goal is not to steer or control reality; instead, *de facto models* aim at capturing reality.

Moreover, ten process mining related activities are identified in [99], which can be grouped into three categories: *cartography* including discover, enhance, and diagnose; *auditing* including detect, check, compare, and promote; *navigation* including explore, predict, and recommend.

Compliance monitoring corresponds to the *detect* activity as defined in [99]: the *de jure* models would serve as representations of the compliance constraints and are hence used to analyze the *pre mortem* logs. Diagnostics are provided to the user if the behavior in the logs is in some way different from the one specified in the models, i.e., detects deviations at runtime (auditing) [99]. An enabling technology for the *detect* activity is *conformance checking*. Conformance checking and related techniques are introduced in Section 3.1.5 together with a discussion on their differences to compliance monitoring approaches.

## 3 Compliance Monitoring Functionality (CMF) elicitation

The elicitation of the CMFs corresponds to *Phase 1* depicted in Fig. 2. To this end, we systematically reviewed literature (Section 3.1) and analyzed five case studies (Section 3.2).

### 3.1 Literature review

After defining the selection criteria, the first input for the elicitation of CMFs is compiled from a systematic literature review. With some adaptations, we follow the procedures for systematic literature reviews as proposed in [16].

#### 3.1.1 Research identification

The search for the primary literature is driven by *RQ1: How to identify compliance monitoring approaches?* (cf. Section 1). *RQ1* can be met by defining the following selection criteria for the search: (a) compliance monitoring on business processes and (b) constraints that refer to process activities. This will be reflected in the keywords searches as well as in processing the literature found.

#### 3.1.2 Selection of primary works (horizontal search):

The search was conducted using scholar.google.com (last access 20 February 2014). In a first step, keywords were searched in the titles of the papers, excluding patents and citations. Table 1 summarizes the results of the horizontal search. It states the searched keywords in the first column, the number of hits in the second, the number of selected papers of the primary search in the third, and the criteria for selecting these papers in the fourth column. If, for example, a paper containing keywords *compliance monitoring* in its title was found, we checked whether this paper refers to business processes as well. Overall, we aimed at finding all papers that combine the aspects *business process*, *compliance*, and *monitoring*. This was important to guide the horizontal literature search while keeping a clear focus. Note that more keywords and combinations were checked than stated in Table 1, however, these keywords and the resulting references are only displayed if at least one paper was found and selected.

Overall, the horizontal literature search resulted in 70 references out of 1605 hits. The respective list of references is available at http://www.wst.univie.ac.at/communities/ComMon/.

#### 3.1.3 Processing of primary literature list

The list of references resulting from the horizontal search was evaluated in a first round excluding papers that

1. are clearly geared towards design time aspects;
2. do not refer to requirements on the compliance specification language;
3. do not refer to constraints at the business process level, but to more low level integrity constraints such as Service Level Agreements (SLAs) or calculating Key Performance Indicators (KPIs).

Obviously, the first filter criterion led us to loose design-time approaches that can be potentially lifted to runtime analysis. This is the case, for example, of [79]. On one hand, we wanted to isolate as sources for our investigation only those approaches that are natively developed for dealing with compliance monitoring. On the other hand, we believe that the CMF framework here presented can serve as the basis for assessing in which design-time approaches are indeed apt to be used at runtime.

For the backward search, the reference lists of the papers presenting a compliance monitoring approach for business processes were analyzed. Moreover, we identified papers that provide surveys on business process compliance approaches such as Becker et al. [28] and validated our search results against these articles by comparing the references. This resulted in adding one reference, namely [18]. Moreover, the backward search led to interesting references in the context of Web Services, i.e., [19,20,24,25,29]. Here it is important to distinguish approaches that concentrate on SLAs (and are not further considered) and approaches that apply compliance monitoring at a process level (i.e., based on a Web Service orchestration) that should be considered. Finally, the backward search resulted in replacing primary paper [30] by more specific papers, i.e., [26,27] as well as primary paper [29] by [21–23]. This step was conducted using the name of the provided tools (MONPOLY in the case of [30] and Dynamo in case [29]) and by going through the papers of the authors. In summary, we extended the primary list by 6−2+5=9 references.

The systematic literature analysis resulted in 60 papers. They were analyzed and synthesized as described in the next section.

#### 3.1.4 Data synthesis

The results from the first and second round of the literature review were analyzed in two rounds; first of all, by assigning each paper to a researcher, followed by a group discussion on all 60 papers. For each paper, it was checked whether it

1. provides a compliance monitoring approach for business processes [17,18,21–24,26,27,31–42],
2. includes studies on process compliance patterns [43–46],
3. provides enabling technologies and related techniques for process compliance monitoring, e.g., conformance checking [47–51],
4. provides frameworks for compliance monitoring infrastructure [52–54] or contract monitoring [55,56], or
5. features domain-specific approaches such as from health care, providing requirements, examples, and case studies [57,58].

Categories 1–5 are processed in the course of the paper as follows (if none of the categories applied then the paper was discarded from further processing):

1. The “core” compliance monitoring approaches in business processes were carefully analyzed and classified using the defined CMFs (see Table 7).
2. The compliance pattern approaches are compared to the CMFF (see Tables 4–6).
3. Enabling technologies and related techniques are discussed in Section 3.1.5.
4. Frameworks for compliance monitoring infrastructure are discussed in Section 3.1.6.
5. Domain-specific approaches are analyzed examples and discussed in Section 3.1.7.

In the remainder we first discuss Categories 3–5, before describing the CFMs. This helps to position our work.

#### 3.1.5 Enabling technologies and related techniques

We briefly survey research approaches that, although not directly focused on compliance monitoring, can be used either as enabling technologies or as techniques for tackling this problem.

At first, relevant line of research is concerned with *conformance checking*. Although the usage of the two terms of *conformance* and *compliance* is not homogeneous in the literature, in the Business Process Management (BPM) setting conformance checking is typically understood as the problem of comparing an existing process model with an event log, so as to understand how far the event log reflects the behavior set out by the process model and, in the case of discrepancies, to measure to what extent they diverge. A number of approaches have been proposed to tackle this problem, see, e.g., [47–49]. The two main differences between conformance checking and compliance monitoring are the kind of model used to analyze the logs (conformance checking usually involves a complete model of the process), and the tackled phase in the process lifecycle (conformance checking is typically applied post mortem) [45]. Despite these two differences, there is a lot of potential in the interaction between these two areas. In particular, observe that many of the conformance checking techniques could be actually lifted to runtime. Furthermore, the fine-grained comparison metrics used in conformance checking to assess how much the input model and log deviate from each other have the potential to extensively contribute to CMF 10 (cf. Section 4.3).

A second group of approaches that are related to compliance monitoring is that of *stream data management*. In broad terms, stream data management focuses on the management of data received as a continuous, real-time sequence of items [96]. The relevance of stream data management for compliance monitoring covers both the querying and the event gathering aspects.

As for querying, part of stream data management deals with query languages, techniques, and tools for stream data, to suitably mediate between the expressiveness of queries and the efficiency of answering. Since such queries are posed against dynamically acquired data, they usually involve temporal operators that can be used to compare and correlate data across time. In this light, those approaches that tackle compliance monitoring by just analyzing the trace of events accumulated so far, without reasoning on the possible future outcomes, can be seen as a special form of query answering over stream data, where the data stream delivers data about the monitored cases, while compliance rules are formulated as special queries. We consider the investigation of these synergies as one of the most interesting lines of research for the future.

As for event gathering, stream data management comes with principles, techniques, and tools for processing a stream of (raw) data produced by multiple, possibly heterogeneous sources, so as to extract, analyze, and infer meaningful events from it. This specific area of research is called *Complex Event Processing* (CEP) [95]. CEP frameworks are able to iteratively clean, refine, correlate, and combine low-level events into abstract, higher-level events. Since compliance monitoring focuses on business-level events, CEP can be considered as an enabling technology for compliance monitoring in all those situations where business-level events are not directly generated by the monitored system, but can be obtained by suitably aggregating low-level events. In addition, CEP can support compliance monitoring in all those large-scale systems where an extremely large amount of events must be analyzed with tight real-time requirements [52–54].

#### 3.1.6 Frameworks for compliance monitoring infrastructure

In this category, we find works that do not propose a specific technical approach for compliance monitoring, but address the problem of implementing a general architecture or infrastructure for compliance monitoring in the literature. These approaches particularly address the challenge of bringing different perspectives of compliance management together. The development of a compliance management architecture is a focus of the COMPAS project. In [97], Mulo et al. propose a systematic method of realizing a compliance monitoring infrastructure in a process-driven SOA. Compliance of a business process instance is determined by monitoring controls applied to the activities of the process. It provides a domain-specific language that enables the definition of single or groups of activities to be monitored. For such activities, conditions for monitoring directives can be defined. The conditions may comprise *filters* to narrow down the amount of particular activity instances that need to be considered by a monitoring component (e.g., only credit worthiness checks with a loan amount exceeding a threshold are to be monitored). Further, conditions are associated with *assertions* that specify expected values of monitored data (e.g., a certain role is expected for credit worthiness checks with a loan amount exceeding a threshold). If assertions are not fulfilled, a compliance control is violated. Being model-driven, the framework further foresees patterns for translating compliance monitoring statements specified in the DSL into code, such as queries, that can be processed by specific compliance monitoring engines. In their prototype implementation, Mulo et al. provide templates for generating queries for the Esper event processing engine. This separates the SOA concerns from the technical compliance monitor and ensures the replaceability of the CEP engine employed. In [52], Awad et al. introduce a framework for implementing an approach addressing compliance monitoring. It is exemplified for Separation of Duty (SoD) requirements how the framework provides support along the process of implementing compliance monitoring from the definition of compliance requirements in controlled natural language to the translation into checkable constraints. The framework relies on CEP for aggregating significant process events (e.g., a completed travel request). Such events may trigger constraints. Constraints are associated with conditions referring to data, resources or roles to be checked (e.g., checking whether SoD is ensured) and actions to be scheduled when conditions apply (e.g., blocking the process execution).

#### 3.1.7 Domain-specific approaches

Middleton et al. [57] and Stevovic et al. [58] address compliance monitoring in the health care domain. In [57], the authors present requirements on providing compliance monitoring functionalities, but more at a technical level such as a common and extensible data model. In [58], the authors utilize business processes as a means to define, implement, and monitor security and privacy policies in sharing Electronic Health Records (EHR). Domain-specific compliance monitoring approaches are not further investigated, but analyzed for examples to illustrate the CMFs.

### 3.2 Harvesting compliance functionalities from selected case studies

Next to our literature review, we used various case studies to assist in the elicitation of CMFs. For harvesting compliance functionalities, in a first step, we analyzed the compliance constraints relevant in five case studies. The case studies were chosen because they cover a diverse set of different domains and we had access to the project data. Table 2 summarizes the details. The number of harvested compliance constraints might seem to be low for some projects at first sight. However, some of the compliance constraints are very complex. For example, the European skin cancer guideline in the “EBMC^2^” project requires a textual description of more than ten pages and entails different CMF functionalities in a single guideline.

## 4 Compliance Monitoring Functionality (CMF) design

This section presents our CMFF, i.e., the framework of *Compliance Monitoring Functionalities* (CMFs). Following the methodology set out in Section 3, we derived CMF candidates from a systematic literature review and five case studies. Based on several rounds of discussions, these candidates were then cleaned and aggregated into the ten CMFs proposed in this paper. Each CMF is described using the following template listing the *name*, a brief *overview* on the CMF, a *description*, guidelines about the *evaluation criteria*, *examples*, and *implementation* hints of compliance rules illustrating their functionality. Whenever possible we directly borrow the examples from literature or the projects. Sometimes we also provide new examples to highlight specific features of the CMFs.

Moreover, the following requirements for CMFs were identified that also serve as basis for classifying the ten presented CMFs in the following (cf. Table 3). Such requirements tackle the three main dimensions of any CMFF: (a) modeling of compliance constraints, (b) analyzing the raw data at runtime, and (c) generating compliance monitoring results to be returned to the end users.

1. *Modeling requirements* A compliance monitoring approach has to enable the specification of compliance constraints that can be monitored. The CMFs of this class refer to the ability of compliance monitoring approaches to express constraints not only on the control flow of a business process, but also on other, equally important, perspectives. This helps in classifying CMFFs with respect to their modeling capabilities, and to position their adequacy in a specific domain with its own compliance constraints to be formalized.
2. *Execution requirements* Compliance monitoring approaches should deal with execution-based information attached to the events of the event stream to be monitored. In general, an event is always related to an activity in a business process, but further information can be also provided. For example, an event can be associated with information related to the activity life cycle or activity data. The CMFs of this class refer to the ability of compliance monitoring approaches to process domain-related information at the event level only available at execution time. As such, even though these requirements are not necessarily tailored to regulatory compliance, they must nevertheless be considered when developing a CMFF. In fact, they characterize the nature of input data to be processed by the monitoring component, and if input data are not understood and analyzed properly, then there is no guarantee about the meaningfulness of the results produced by the monitoring facility. For instance, if the input data are events tracking the execution of non-atomic activities, the monitor must be able to reconstruct the notion of activity properly from the raw, processed events.
3. *User requirements* The third dimensions focus on the ability to return the compliance assessment to the end users. Specifically, *advanced diagnostics and recommendations* relate to the ability of a CMFF to provide advanced, meaningful information to end users that go beyond violation detection and explanation. For example, it could be useful for the end-users to not only know why a certain rule has been violated, but also what they should have done instead in order to correctly continue the execution. If this kind of analysis is done *after* a violation has taken place, then we classify it as a form of *advanced diagnostics*. If, instead, the analysis is carried *a priori*, to suggest measures for preventing inevitable violations, then we talk about *pro-active recommendations*. Clearly, the latter is particularly relevant when a compliance rule is still in a violable state.

### 4.1 Modeling requirements

The following three CMFs refer to the ability of a compliance monitoring approach to deal with constraints that address aspects beyond control flow: time, data, and resources.

*CMF* 1: *Constraints referring to time.*

*Overview*: The bulk of real-world process compliance rules involves a combination of multiple activities or events in time. Hence, time is obviously one of the most important dimensions that a compliance rule language must tackle.

*Description*: Time-related conditions within compliance monitoring constraints may be *qualitative* or *quantitative* (i.e., metric time). This determines how temporal entities can be related to each other. A qualitative notion of time supports the comparison between temporal entities without referring to their actual distance. Typical qualitative temporal patterns are “before” and “after”. Such temporal relations are utilized, for example, to capture the fundamental ordering between events constrained by a compliance rule. In contrast to qualitative time constraints, metric (or quantitative) time constraints specify the distance between time entities. Metric constraints typically refer to deadlines, delays and latency constraints in compliance rules [14,39].

*Evaluation criteria*: To fully support this functionality, the approach must be able to monitor qualitative *and* quantitative time-related conditions.

Examples:

• (Qualitative time) For payment runs with amounts beyond €10,000, the payment list has to be signed before being transferred to the bank and has to be filed afterwards for later audits [35].
• (Qualitative time) When an investor receives an amount of money, she becomes in charge of eventually investing it in bonds or in stocks and she cannot receive money anymore before the investment. [37].
• (Quantitative time) For Stage 1A patients, an appointment for sonography has to be made within 12 months (European Skin Cancer Treatment guideline [60]).
• (Quantitative time) A passenger ship leaving Amsterdam has to moor in Newcastle within 16 h [63].
• (Quantitative) If employing any electronic storage media other than optical disk technology (including CD-ROM), the member, broker, or dealer must notify its designated examining authority at least 90 days prior to employing such storage media [33].

*Implementation*: We briefly discuss the case of atomic timestamps, which are associated to a point-based algebra (see CMF 4 for a discussion on durative time entities). Temporal logics such as LTL, CTL* and *μ*-calculus [65] all adopt an inherent qualitative notion of time. Thus, they easily capture qualitative temporal relations such as “before” or “after”. If not already inherent, such temporal relations can be introduced to the compliance rule language as the semantics of these relations can be defined over execution traces (and their linear/branching future). When metric times come into play, two approaches are typically followed for their representation: an *implicit* approach, embedding them inside temporal operators (like in real-time logics such as MTL and TLTL [66]) or an *explicit* approach, where explicit time variables are introduced and subject to arithmetic constraints (like in extensions of logic programming such as the Event Calculus [67]).

*CMF* 2: *Constraints referring to data*

*Overview*: Compliance rules often not only define constraints on activities or events but also contain conditions on data processed in a business process.

*Description*: Data refer to the ability of the compliance rule language to not only target the control-flow aspect, but also the *data aspect*. This leads to data-aware compliance rules that can include constraints, requirements and expectations about data objects and their values.

For what concerns the constraints׳ shape, a major distinction can be drawn between *unary data conditions* that just involve a single data object and *extended conditions* that possibly relate multiple data objects at the same time. Unary data conditions take the form d⊙v, where *d* is some data object, ⊙ is a comparison operator and *v* is some value of *d*׳s domain. Extended data conditions express comparisons between multiple data objects, e.g., comparing the values of data element *temperature* measured at two different activities within a business process. According to the classical data-related workflow patterns [68], we can further distinguish between different sources of data, namely *activity data*, i.e., data taken as input or produced by the activities of a business process and *case data*, namely data that are associated to a whole process instance and can be accessed/manipulated by all activity instances executed inside the case.^1^

*Evaluation criteria*: To fully support this functionality, the approach must be able to monitor unary data conditions *and* extended data conditions over activity *and* case data.

Examples:

• (Activity data) If the PainScore of patient *p* is greater than 7 and the status is uninitialized then the status must be changed to initialized and a timer event is generated to treat patient *p* within 1 h. (This rule is based on a formal description in [57].)
• (Case data/extended data condition) If a vessel (case) is of type fishing boat, the size of the boat is above 25 m (100 tons) and it is located at 54° of latitude and 8.5° of longitude, it cannot be engaged in fishing [63].
• (Unary data condition) In case the total number of users permissible on the server has reached the limit, access privilege to current potential user requires exception approval from IT administrator [40].
• (Comparison of multiple data objects) If the first test terminates with a particular result code, then all the consequent executions of the test should return the same result code.
• (Comparison of multiple data objects) Any [LightPathOperation (LPO)] ID appearing in any partition request must be different from any LPO ID appearing in any future concatenate request [24].

*Implementation*: Data-aware compliance rule languages typically employ variables to denote data objects and conditions to pose constraints over them. The main difference lies then in the domains of the data objects, as well as in the “shape” of such constraints. In order to support data-aware compliance rules, the corresponding compliance monitoring approach must be able to evaluate the truth of data conditions. This necessitates access to respective data sources within the process runtime environment.

*CMF* 3: Constraints referring to resources

*Overview*: Compliance constraints often relate to organizational resources involved in the business process.

*Description*: Compliance rules often involve not only the control-flow and the data perspective but also the organizational perspective of a business process. This is particularly true for compliance rules stemming from legal sources. Resource-related conditions in compliance rules can be considered a special case of data-related constraints where the data refers to the resources involved. This is because resource-related information is often represented as case or activity data. Resource-aware compliance rules include constraints, requirements and expectations on resources (e.g., agents or roles) ass;ociated with activities or events. Similar to data-related constraints, we can distinguish between *unary resource conditions* expressing expectations on specific resource properties in isolation and *extended resource conditions* relating multiple resources.

*Evaluation criteria*: To support this functionality, the approach must be able to monitor unary resource conditions *and* extended resource conditions.

Examples:

• (Unary resource condition) The bank must verify the identity of each customer, using the information obtained in accordance with the above requirements, within a reasonable time after the account is opened [33].
• (Unary resource condition) Orders of more than 1000€ *can only be approved by a senior manager.*
• (Extended resource condition) Final approval of the assessment can only be granted by the manager that requested the assessment.
• (Extended resource condition) Every closed project must be validated by a person who did not participate in the project. (*4-Eyes principle*, also called Separation of Duties (SoD).)

*Implementation*: Depending on the particular event model and the process runtime environment, constraints on resources may be dealt with in a similar manner as data-related constraints. Clearly, the evaluation of resource-related constraints requires access to resource information (such as originators, roles, groups) during process execution. This is supported by the XES organizational extension [7] (cf. Section 1).

### 4.2 Execution requirements

There are several requirements imposed on compliance monitoring approaches by the domain. The following CMFs enable the assessment whether or not a compliance monitoring approach meets these requirements.

*CMF* 4: *Supporting non-atomic activities*

*Overview*: Activities in a process may be non-atomic, i.e., may have a duration. Hence, compliance rule languages must also support non-atomic activities.

*Description*: *Non-atomic activities* are durative activities whose execution spans across a time interval. While the execution of an atomic activity is associated to just a single event attesting that an instance of the activity has been “done”, non-atomic activities are associated to multiple events and to a lifecycle that disciplines the allowed orderings among such events. The lifecycle contains at least the two event types *start* and *complete*. Moreover, often additional event types such as *suspend, resume, abort* are possible [8]. Compliance rules dealing with non-atomic activities follow either an *explicit* approach, talking about their multiple, atomic constitutive events, or an *implicit* approach, where the activities are mentioned as such without referring to their events. If the approach is explicit, the definition of compliance rules is similar to what can be done with atomic activities (now mentioning the atomic constitutive events of each activity). However, if the approach is implicit it is necessary to match the implicit semantics of the rules with the information provided in the actual data where activities are distributed over multiple events.

*Evaluation criteria*: To fully support this functionality, the approach must be able to monitor explicit *or* implicit conditions on non-atomic activities.

Examples:

• (Explicit) An order creation cannot be completed until the customer registration is completed.
• (Implicit)Activity check project can be executed only while the project is under preparation.
• (Implicit) Activities First Medical History and Excision Melanoma must not overlap [59].

*Implementation*: Implementations differ depending on whether the explicit or implicit approach is adopted. With the explicit approach, the monitoring framework must be able to handle at least two types of information about each event: the activity it refers to and its type, which must be one of the event types constituting the activity lifecycle (start, complete, suspend, resume, abort, etc.). Since the language directly tackles these constitutive atomic events, it typically relies on a *point-based algebra* to relate their relative position in time. The implicit approach directly targets activities and assumes that the time windows corresponding to their (non-atomic) executions can be reconstructed from the monitored event stream. Since the compliance rule language predicates in this case over durative temporal entities, it relies on an *interval algebra* (such as the one by Allen [69]) to relate the execution of different activities over time. See [70] for a survey on temporal reasoning.

*CMF* 5: *Supporting activity lifecycles*

*Overview*: Non-atomic activities are associated with a lifecycle defining the allowed orderings of the constitutive events. Suitable monitoring mechanisms should be provided to check whether this lifecycle is indeed followed.

*Description*: The activity lifecycle describes the allowed executions of atomic, correlated events that together describe the execution of non-atomic activities over time. In particular, the lifecycle lists the states in which an (instance of a) non-atomic activity can be at a given time, the constitutive events that mark a step in the execution, as well as in which states such events may happen, and to which state they lead. This latter aspect implicitly defines the allowed orderings of the constitutive events. The lifecycle is therefore mostly captured by a state chart (cf. ADEPT [71] or iUPC [72]). In general, multiple, independent executions of the same activity (i.e., activity instances) can occur inside a case. Each such instance corresponds to an instance of the activity lifecycle. A proper *correlation* mechanism is required to correctly manage the progressions of each lifecycle instance and, in particular, to associate a given event to the right corresponding lifecycle instance. For example, if two starts of some activity and two completions of the same activity occur during a case, it is necessary to identify to which start event each completion event refers. From the monitoring point of view, (meta-)rules capturing the activity lifecycle and its instances can be used to check whether the activity executions contained in a given trace indeed comply with the expected lifecycle constraints.

*Evaluation criteria*: To fully support this functionality, the approach under study must capture the activity lifecycle *and* implement a correlation mechanism between events. Note that the correlation of activity instances builds on the correlation of process instances (cases). Within the same process instance, there may be multiple instances of the same activity.

Examples:

• (Activation) *A start event creates an activity instance and puts it into the active state* [7,9].
• (Completion) *Each completion event moves its associated activity instance to the completed state, provided that the instance is currently active* [7,9].
• (Balance start/complete events) *For every activity instance, each start event has a single corresponding completion or cancelation event* [7,9].

*Implementation*: Implementations of this CMF are possible if the compliance rule language supports:

1. the notion of “state”, and
2. a correlation mechanism between events.

Out-of-order events can either be ignored, or managed by putting the corresponding activity instance into a special “error” state, pointing out that a deviation from the expected lifecycle has been detected. Correlation can be realized by providing a special parameter used to identify the corresponding activity instance. This way, two events carrying the same identifier are recognized to be part of the same lifecycle. Events carrying different identifiers but referring to the same activity correspond to potentially parallel lifecycle instances.

*CMF* 6: *Supporting multiple-instances constraints*

*Overview*: There may be multiple instances of the same compliance rule in a trace due to multiple, possibly parallel occurrences of the involved activities. Monitoring at the constraint instance level allows for tracking fine-grained compliance rules.

*Description*: When compliance rules are able to express requirements about time (CMF 1), data (CMF 2), and/or resources (CMF 3), the same compliance rule can be activated multiple times, as multiple events referring to the activities targeted by the rule occur, each with its own timestamp, data and resource information. In fact, each of such events provides a specific “context” for the compliance rule. This context is then used to instantiate the temporal/data/resource conditions possibly associated with the compliance rule. Consider, for example, the rule stating that *every time an order O is closed by the client, then order O must be eventually delivered by the warehouse*. Clearly, the constraint is instantiated for each specific closed order and each instance has its own evolution depending on events specific for this order. For example, it could happen that two orders are closed but only one is delivered. In this case, two instances of the compliance rule should be generated by the monitoring framework, then judging one of them as satisfied and the other one as violated. A more detailed discussion on multiple instances handling can be found in [35,39].

*Evaluation criteria*: To support this functionality, the approach must be able to monitor multiple instances, where the notion of instance is substantiated using temporal *and/or* data *and/or* resource-based conditions.

Examples:

• (Multiple instances based on timestamps) Every final submission has to be corrected within 6 weeks. Here, every submission cycle termination creates an instance of the compliance rule determined by its time stamp *t*; the instance then checks that the correction occurs between *t* and t+6, assuming a granularity of weeks [62].
• (Multiple instances based on data and resources) The carbon footprint of a supplier must not exceed a value of *x*. Depending on the number of suppliers modeled as resources, the constraint is instantiated multiple times. If suppliers can be added during runtime, the number of constraint instantiations will increase accordingly. (The carbon footprint is one of the non-functional optimization parameters in the ADVENTURE project [98]. The rule can be derived from the combination of optimization of multiple process instances with dynamic selection of partners as provided in ADVENTURE).

*Implementation*: Supporting multiple instances of a compliance rule requires mechanisms to discriminate between different rule activations. This can be achieved by precisely characterizing which information (time, data, resources) contributes to define the “context” of the rule (see the examples below) and which are the events that create separate instances of the rule by filling this context with specific values. Each observed rule instance has to be associated with a separate compliance state in order to assess compliance at the rule-instance level.

### 4.3 User requirements

The CMFs described in this section refer to the ability of a compliance monitoring approach to address user requirements.

*CMF* 7: *Ability to reactively detect and manage compliance violations*

*Overview*: If a violation is detected by the compliance monitoring approach, it could simply report it and provide no further support. Once the behavior is non-compliant and this is irreversible, the monitor may take the viewpoint that no further support is needed. However, a compliance monitoring approach may accommodate a variety of additional advanced features (besides detection) to continue the monitoring after a violation takes place, give feedback to the user and suggest compensation actions.

*Description*: In the context of reactive detection and management of compliance violations, these factors can be exploited to characterize the degree of support provided by a compliance monitoring approach:

• *Detection*, the ability to detect compliance violations.
• *Feedback*, the ability to provide detailed compliance reports (see CMFs 9 and 10).
• *Continuous monitoring*, the ability to continue monitoring after a violation.
• *Recovery and compensation* mechanisms, used to react to a violation with proper countermeasures.

*Evaluation criteria*: To support this functionality, first of all, the approach must be able to detect a compliance violation and provide an intelligible compliance report. In addition, the approach should be able to guarantee continuous monitoring, i.e., it should be able to continue the compliance monitoring after the violation has happened. This can be supported, for example, by implementing recovery and compensation mechanisms in the case of compliance violations. This can comprise automatic and semi-automatic treatment of violations.

Examples:

• (Continuous monitoring) Generally, the patient has to formally confirm that she has been informed about risks prior to invasive treatments. If this is not the case (e.g., in emergency cases), this has to be documented and the patient has to be informed about the treatment risks afterwards (*contrary-to-duty* obligation). This constraint requires the ability of continuing the monitoring, even after the compliance constraint has been violated by not informing the patient prior to the invasive treatment.
• If the PainScore of patient *p* is greater than 7 and the status has not been initialized yet, then the status must be changed to initialized and a timer event is generated to treat patient *p* within 1 h. If there is no response within 1 h, a response warning is sent and another timer event for 1 h is set. (This rule is based on a formal description in [57]). The example shows that even if the compliance constraint is violated for the first time, a strategy for continuing the monitoring is defined.)

*Implementation*: As for recovery and compensation, an added feature of the compliance rule language is the ability of dealing with violations. An event violating a rule can be used to contextualize it, making the rule active only when some violation takes place. This kind of rule represents a form of recovery or compensation, which introduces further constraints/requirements upon a violation. This can be realized, for example, by introducing notions like *contrary-to-duty* operators [73] or *reparation chains* [79] in the compliance rule language. Approaches that query the partial execution trace for certain event patterns, such as [42,35], typically do not have difficulties with continuing after detecting a violation. However, continuous monitoring can be a challenge for logic-based approaches (e.g., [74]) as the approach must be able to tolerate inconsistencies to continue monitoring after a violation occurred. In [37], the authors introduce some recovery capabilities to realize different strategies for continuous monitoring showing that automata-based approach are also able to accommodate sophisticated recovery mechanisms.

*CMF* 8: *Ability to pro-actively detect and manage violations*

*Overview*: While recovery and compensation measures may be applied when detecting a violation, the violation itself cannot be undone. To prevent possibly costly compensation on non-compliance, a compliance monitoring approach should be able to provide support to pro-actively detect and manage possible compliance violations.

*Description*: Pro-active support includes detecting possible and unavoidable future violations and mechanisms for preventing violations. Future violations are violations whose source is not yet explicitly contained in the trace. They can be detected by implicit violations caused by currently conflicting rules. The presence of conflicting rules identifies violations that cannot be revealed by considering each compliance rule in isolation, but only by merging the contribution of two or more compliance rules. The early detection of such future compliance violations enables timely preparation of recovery and compensation actions. Support for preventing violations refers to the ability of a compliance-monitoring framework to provide assistance for complying with imposed rules before compliance violations become manifest. This comprises, for example, predictions and recommendations of activities to be executed next in order to preserve compliance.

*Evaluation criteria*: To support this functionality, the approach must implement mechanisms for the early detection of conflicting conditions *or* provide the user with recommendations about what to do next to avoid violations.

Examples:

• (Early detection of a violation) Every time an order is delivered, the warehouse must be replenished. If the replenishment truck is broken, the warehouse cannot be replenished. Consider an execution where the truck is broken and the order delivered. Approaches able to detect conflicts among rules would in this case point out an (implicit) violation: the first constraint requires a replenishment and the second forbids it.
• (Proactive support to comply) Conducting a payment run creates a payment list containing multiple items that must be transferred to the bank. Then, the bank statement must be checked for payment of the corresponding items. For payment runs with amount beyond 10,000 €, the payment list has to be signed before being transferred to the bank and has to be filed afterwards for later audits. For a concrete payment run with an amount beyond 10,000€, the monitoring system can deduce from the constraints that two activities (namely *sign the payment list* and *file the payment list*) are pending and need to be executed to comply. This can be exploited for ensuring that the pending tasks are scheduled and for preventing the transfer of the payment list to the bank unless it has been signed.
• (Predictions and recommendations) Requests for building permits need to be handled within 3 months. Based on historic information, i.e., comparing a request currently being handled with earlier requests, one can predict the remaining processing time. A counter measure is taken if the predicted remaining processing time is too long.

*Implementation*: Future violations as described can be detected when considering the interaction of all imposed compliance rules. A typical task is evaluating whether the compliance rules are not conflicting a priori, i.e., that the whole set of rules admits at least one compliant execution trace.

However, compliance rules that are not conflicting in general may still become conflicting at some point during the process execution. Thus, checking of compliance rules at design-time or per individual constraint, is not sufficient for detecting all types of future violations. It should be noted that supporting such implicit violations can become quite costly and the cost grows with the amount of rules involved. For expressive compliance rule languages, this becomes even undecidable.^2^ The identification of suitable decidable compliance rule patterns for data- and time-aware compliance rules is still an open challenge.

To avoid violations in a running process instance, it is also possible to give recommendations about what to do next by exploiting complete cases stored in event logs (e.g., using process mining techniques [99]) or by analyzing the prevailing obligations to satisfy compliance rules.

*CMF* 9: *Ability to explain the root cause of a violation*

*Overview*: Key to the practical application of a compliance monitoring approach is its ability to pinpoint the root cause of a compliance violation beyond providing the counterexample that resulted in the violation. This is particularly true when a compliance rule can be violated in multiple ways or multiple rules are involved in a violation.

*Description*: *Root-cause analysis* enables to diagnose the root cause of a compliance violation, e.g., by isolating the responsible event occurrences or the involved compliance rules. Note that this kind of analysis is far from trivial and sometimes could lead to multiple possible explanations or to no explanation at all. Consider, for example, the case of a sequence of events that culminates in the expiration of a deadline: isolating the responsible events in this case is impossible in general. Similarly, as discussed in [38] there can be multiple sets of compliance rules that are involved in a violation at the same time and therefore fine-grained analysis is needed to identify the minimal set(s) of conflicting rules. Beside the root cause analysis itself, it is also of utmost importance to provide suitable ways for communicating the result of the analysis to the end users in a comprehensible and intuitive manner [35].

*Evaluation criteria*: To support this functionality, the approach must implement mechanisms for root cause analysis and support their effective communication.

Examples:

• (Root-cause of a violation within one rule) When a patient is diagnosed with cryptorchidism, an operation must be performed either through laparoscopy or with an open surgery but not both. This rule can be violated in two different ways (can have two different root-causes), i.e., no operation is performed or both laparoscopy and open surgery are performed in the same case.
• (Root-cause of a violation involving multiple rules) Typically each warehouse order undergoes a sequence of three steps: preparation, packaging, transportation via a conveyer belt. A domain constraint states that when the conveyer belt breaks, it cannot accomplish the transportation task anymore. Even though there is no direct incompatibility between the “belt broken” event and the preparation of the order, if both occur, this will cause a violation.

*Implementation*: For future research, efforts should be taken to provide diagnostics and pro-active recommendations based on the identification of the root cause of a violation. So far this is only supported by few approaches [35,38,75].

*CMF* 10: *Ability to quantify the degree of compliance*

*Overview*: Compliance metrics and indicators should be employed by a monitoring framework to provide aggregated feedback to the users, summarizing the detailed information computed for each compliance rule.

*Description*: The practical feasibility of a compliance monitoring approach also relies on its ability to give practitioners a sense of the compliance situation. For that, crisp approaches associating two possible truth values to each compliance rule, representing whether it is satisfied or violated, is not sufficient. In contrast to crisp compliance characterization, fuzzy approaches allow for a range of values to capture the “degree of compliance” of the running trace with respect to a compliance rule. In this respect, we differentiate between approaches that *discretize* the possible truth values from approaches that adopt *continuous* distributions between 0 (violation) and 1 (satisfaction).

*Evaluation criteria*: To support this functionality, the approach must be able to characterize the “healthiness” of a running trace through metrics. Advanced support is provided by an approach that is able to derive statements about the health of the whole process/system by evaluating multiple running traces, i.e., by determining the fraction of deviating traces.

Examples:

• (Metrics) A vessel cannot be not under command, a vessel with one occurrence of not under command is more “healthy” than a vessel with nine occurrences of not under command [63].
• (Fuzzy) A passenger ship leaving Amsterdam has to moor in Newcastle within 16 h. It is desirable to judge with different degrees of violation a ship arriving in Newcastle after 16 h and 10 min and a ship arriving in Newcastle after 18 h [63].

*Implementation*: A typical approach to quantify the degree of compliance is to “count” the number of violations and devise meaningful metrics that give a measure about the *overall compliance degree* of a running process instance. This is particularly effective when multiple instances are managed (cf. CMF 6). More fine-grained metrics can be devised by using detailed information about individual violations. Approaches using a continuous scale need to calculate a “degree” of compliance, rather than simply providing a yes/no answer. For example, in the case of a deadline, a matching function could assign different noncompliance weights to traces missing the deadline, depending on the amount of time that passed between the deadline and the (late) event occurrence.

## 5 Compliance Monitoring Functionality (CMF) realization

This section is concerned with *Phase 3 CMF Realization* of the methodology depicted in Fig. 2. Phase 3 consists of three building blocks, i.e., a pattern-based comparison of CMFs related to language aspects with compliance patterns set out in the literature (cf. Section 5.1), a classification of existing monitoring approaches using the CMF framework (cf. Section 5.2), and the application of the CMFF in selected tools (cf. Section 5.3).

### 5.1 Common compliance rule patterns and the CMF framework

We provide a brief, comparative survey about some of the most typical compliance rule patterns found in the literature, so as to concretely substantiate the relevance of the language-related CMFs, namely CMFs 1–4. It is worth noting that the vast majority of the literature takes inspiration, for such patterns, on a catalog of temporal logic specifications typically employed in model checking [76]. The semantics of such patterns are not exactly the same, though: while [76] follows the standard infinite-trace semantics for dynamic systems, compliance rule patterns are typically meant to be checked against a partial trace whose continuation will be finite.^3^

Specifically, Tables 4 and 5 summarize typical patterns related to the control-flow dimension of compliance rules. The tables respectively tackle the (co-)occurrence and relative orderings between activity executions, substantiating the need for qualitative time constraints (cf. CMF 1). In fact, each of the listed patterns express requirements on the expected/forbidden execution of atomic activities over time, but without expressing metric constraints over the corresponding timestamps. Extensions of patterns in Table 5 with metric time have been studied in [39,74,81,82], supporting the need for quantitative time constraints as well (cf. CMF 1 again).

Furthermore, it is not surprising that such patterns have been also extended with non-atomic activities (cf. CMF 4), given how much widespread they are in BPM [80,74,39]. Data (cf. CMF 2) have been also considered in combination with patterns of the forms shown in Tables 4 and 5, see [39,83–86]. Differently from data and non-atomic activities, dedicated resource-related patterns (cf. CMF 3) are mentioned in [43,82]. It is interesting to notice that, as argued in CMF 3, not only *unary resource conditions*, but also *extended resource conditions* are present. Extended conditions are in fact necessary to relate and compare performers of different activities in a compliance rule.^4^

Finally, observe that, from the expressiveness point of view, the resource patterns in Table 6 can be re-expressed using the data-aware extensions of [39,84–86]. This can be done by introducing special data slots tracking activity originators, and adding specific conditions on them. However, from the modeling perspective data and resources have a different nature. This is why they are separately tackled by two different CMFs.

### 5.2 Classification of compliance monitoring approaches

We classified compliance monitoring approaches using the ten CMFs presented in this paper. We focused on compliance monitoring approaches that mainly address compliance checks during the process execution. These approaches are different from other approaches that can be used in other phases of the process lifecycle such as process design (e.g., [90]) or compliance constraint modeling (e.g., [1,91]) and trigger specific questions. For example, monitoring is carried out with actual data and by considering finite, evolving prefixes of event traces.

Furthermore, we selected the approaches to be classified based on the degree of detail on concepts provided in publications. In fact, a certain degree of detail (for example, in the used compliance rule languages) is necessary to properly classify the approaches through our framework.

The results of the classification are shown in Table 7, where “−”, “+” and “+/−” indicate functionalities that are not supported, supported and partially supported from the conceptual viewpoint, respectively. A rating of “n.a.” indicates that the CMF cannot be assessed based on the analyzed literature. The scores shown in bold refer to approaches where the implementation is *publicly* available.

The first ten approaches as summarized in Table 7 are discussed in this section. The approaches proposed by the authors, namely *MobuconEC*, *MobuconLTL*, and *SeaFlows*, will be described in detail in Section 5.3 in order to showcase the implementation of CMFs along two data sets.

The detailed description of the classification results starts with the framework described in [17] which is based on Supervisory Control Theory. This approach allows for the definition of constraints on resources but, in general, it does not support data conditions. Through this approach, it is possible to supervise the process-aware information system by “blocking” those events that would lead to a violation. This can be considered as a very sophisticated form of pro-active violation management, which is applicable only when the process-aware information system can be (at least partially) controlled by the monitor. Since violations are prevented, the framework does not directly consider the problem of reactive management nor violation explanation.

ECE rules [31] are a domain-independent approach that was not specifically tailored for business process monitoring. Therefore, functionalities like support for case data and activity life cycle were simply not investigated (this is matter of ongoing work). ECE rules can deal with both atomic and non-atomic temporal entities, capturing qualitative and metric time constraints, as well as point-based and interval-based ones. Two key features characterize ECE rules. First, they support an imperfect (i.e., fuzzy and probabilistic) matching between expected and occurred events and hence deal with several fine-grained degrees of compliance. Second, expected events can be decorated with countermeasures to be taken in the case of a violation, hence providing first-class support for compensation mechanisms.

With BPath [42], Sebahi proposes an approach for querying execution traces based on XPath. BPath implements a fragment of first-order hybrid logic and enables data-aware and resource-aware constraints. Quantitative time is supported by referring to and comparing timestamps of events. This enables sophisticated time constraints. Due to the querying nature of this approach, it is able to distinguish between multiple activations of the same compliance rule. However, the approach still lacks support for advanced diagnostics and pro-active compliance management. These issues do not seem to be in the focus of the work. A compliance degree could be calculated from the results provided by this approach. The prototypical implementation of BPath is also presented in [42].

An approach based on constraint programming is provided in [34]. As time aspects play a crucial role in this approach, CMF 1 is a focal issue in this approach. The approach also explicitly addresses the duration of activities. The main goal of [34] is to pro-actively detect compliance violations and to explain the root cause of the violation. The constraint satisfaction problem for the example provided in the paper has been implemented. However, there is no prototypical implementation of a compliance-monitoring framework.

Giblin et al. [33] present an approach based on Timed Propositional Temporal Logic for transforming high level regulatory constraints into REALM constraints that can be monitored during process runtime. [33] explicitly elaborates on temporal constraints, hence addressing CMF 1. As the main focus of the paper is on the transformation and correlation, the other CMFs cannot be evaluated upon this paper. The paper presents architectural considerations and an implementation of the transformation on the basis of a case study.

Narendra et al. [40] address the problem of continuous compliance monitoring, i.e., they evaluate the compliance of a process execution with respect to a set of policies at runtime. In particular, the policies are checked when some specific tasks are executed, which are called control points. The authors define an optimization problem to find the right balance between the accuracy of the compliance checking and the number of control points (each control point has a verification cost). They define policies in terms of first order clauses and their framework can support data- and resource-based rules. There is no notion of time. The framework supports a reactive violation management and not a pro-active violation management. The ability of the framework to detect the root cause of a violation cannot be assessed based on this paper. The authors, however, define metrics to evaluate the degree of compliance of a process instance.

Thullner et al. [41] define a framework for compliance monitoring able to detect violation and to suggest possible recovery actions after the violation has occurred. The framework is focused on the detection of different types of time constraints. A violation is handled after it has occurred and, in this sense, the framework support CMF 7 but not CMF 8. The other CMFs cannot be assessed based on this paper.

MONPOLY [26,27] is a runtime verification framework for security policies, specified in the logic MFOTL, a variant of LTL-FO with metric time. Monitorable formulas are those of the form “Always Φ”, where Φ is a so-called *bounded* MFOTL formula, i.e., a MFOTL formula that can be evaluated within a bounded number of steps in the future. The high expressiveness of MFOTL allows one to express sophisticated compliance rules involving advanced temporal constraints, as well as data- and resource-related conditions. Consequently, even though non-atomic activities and their lifecycle are not explicitly tackled by the approach, they could be properly accommodated. The monitoring algorithm, described in [27] and implemented in the publicly available MONPOLY tool [26], supports continuous monitoring, in that it fetches all the time points at which a violation is detected. Due to the high expressiveness of MFOTL, no advanced features related to pro-active management of violations can be supported.^5^

Halle et al. [24] provide an approach based on LTL-FO^+^. “LTL-FO^+^ is a linear temporal logic augmented with full first-order quantification over the data inside a trace of XML messages” [24] that focuses on monitoring data-aware constraints over business processes. The data is part of the messages that are exchanged between the process activities. The approach can handle unary and binary data conditions. As time and resources can be handled based on data, the approach is also able to deal with CMF 1 and CMF 3, although these CMFs are not explicitly mentioned in the paper. Based on [24], CMF 4–10 cannot be assessed.

The work by Baresi et al. on Dynamo [22] constitutes one of the few techniques and tools dealing with monitoring (BPEL) Web Services against complex rules, going beyond the analysis of quantitative KPIs for service-level agreement. In Table 7, we use (Timed) Dynamo to identify the combination between Dynamo and the following languages: timed WSCoL [21] to specify the rules to be monitored, and WSReL to specify the recovery mechanisms to be put in place when given violations are detected [23]. (Timed) Dynamo allows one to model ECA-like rules that mix information about the location (i.e., the target Web Services and operations), the involved data, and qualitative/quantitative temporal constraints. Location-related aspects can be considered as referring to resources in a business context, but since the focus is on interacting Web Services, there is no support for more advanced resource-related aspects such as groups and roles. Timed WSCoL supports the correlation between messages, and is thus able to deal with non-atomic activities, but being focused on Web Service message exchange, no notion of activity lifecycle is considered. Dynamo provides continuous, reactive monitoring facilities based on the messages fetched so far, with sophisticated recovery and compensation mechanisms based on WSReL. Furthermore, the monitoring results can be aggregated and reported to the user, providing useful insights that go beyond a yes/no answer.

In [18], Namiri et al. describe an approach that is based on the patterns proposed by Dwyer and Corbett [76]. In particular, the authors introduce a set of control patterns. Control patterns are triggered by events (such as the execution of a controlled activity). When being trigged, conditions associated with the control are evaluated. In order to provide data for evaluating the associated conditions, Namiri et al. introduce a semantic mirror that is filled with runtime data of a process instance. For each control, actions to be carried out if a control fails may be specified (CMF 7). However, root cause analysis (CMF 9) and pro-active support (CMF 8) are not addressed. Being based on [76], the approach is restricted to a predefined set of patterns. Data (CMF 2) and resource (CMF 3) constraints are supported as conditions to be evaluated once a control becomes triggered. This corresponds to evaluating a query on process data. Metric time (CMF 1) is, however, not addressed. Furthermore, the approach does not incorporate a notion of activity lifecycle (CMF 5). As events may occur multiple times within a process execution, there may be multiple activations of a control (CMF 6). Using the semantic mirror, non-atomic events (CMF 4) can be supported using this approach. An implementation of the approach is also described in [18]. Specifically, controls are implemented as ECA rules, which are evaluated by Drools.

As can be seen from Table 7, the majority of approaches focus on time aspects (CMF 1) and reactive management of compliance violations (CMF 7). This is not astonishing since time constitutes an important requirement in many application domains. In this paper, we have looked at constraints from the health care, the financial, and the maritime safety domain. However, in many other domains the adherence of time constraints and their violation is an important matter, e.g., in logistics. Intuitively, each approach should support at least one of CMF 7 and 8, i.e., provide reactive and / or proactive management of compliance violations. Interestingly, Supervisory Control Theory [17] is the only approach that supports the proactive management, but not the reactive management of compliance violations. This is the case since events that will lead to violations (proactive) are blocked before they actually occur. All other approaches enable the detection of violations when they occur (reactive).

Overall, it can be stated that there are approaches that can deal with modeling requirements, i.e., incorporate time, data, and resource aspects. When it comes to execution requirements such as supporting multiple constraint instances, less approaches are available that support all of the related CMFs, i.e., CMF 4–6. However, the least number of approaches addresses user requirements such as CMF 7–10. For example, only few approaches enable the explanation of the root cause of a compliance violation. As the user requirements are often of particular importance, e.g., if medical staff has to react on compliance violations in stressful situations, it would be desirable to put more research effort on user feedback and support in dealing with compliance violations during run time.

### 5.3 Application of CMFs in selected tools

In Section 5.2, we classified existing compliance monitoring approaches using the CMFF. This was done based on the information in publications. For approaches assigned a “+” or “+/−” for a CMF, the concrete implementation of this CMF may still vary. Thus, it will be interesting to also have a look at the application of CMFs in the tool implementations. The authors of this paper proposed three approaches, namely *MobuconEC*, *MobuconLTL*, and *SeaFlows*, that make use of different techniques to enable compliance monitoring. In the following, we showcase the application of the ten CMFs in these three tools. As these are the tools we know best, it is ensured that we are able to correctly apply the tools in a case study for discussing the implementation of CMFs.

In order to analyze tools using the CMFs, a data set consisting of compliance rules covering the CMFs to be investigated and process instances (or process logs for replaying process instances) are necessary. We selected the Business Process Intelligence Challenge (BPIC) data sets from 2011 and 2012. Using the process-mining tool ProM,^6^ we were able to derive, from these data sets, a set of compliance rules that covers all language-related CMFs. To apply the CMFs, we applied the tools to check compliance of the process instance logs with the set of rules derived the BPIC data.

Since the BPIC data sets contain post-mortem data about complete execution traces, we used a “log replay” component so as to feed the tools with an evolving stream of data that recreate the real executions. This technique makes post-mortem data indistinguishable from truly runtime data. Notice that our aim is to assess the tool functionalities in relationship with the CMFs, and not to go into the details of non-functional aspects such as reaction time and performance-related insights. For such details, please refer to [38,39].

#### 5.3.1 Data set

Since 2011, the BPI Workshop features an initiative called *International Business Process Intelligence Challenge* (BPIC). The idea is that an event log is provided with some background information and points of interest. Researchers and practitioners participate in a competition in which they are asked to test, apply or validate whatever technique or tool they developed using this log. In 2010, the three universities of technology in The Netherlands joined forces in erecting the 3TU Datacenter. This initiative aimed at publicly sharing datasets such that other researchers can benefit from whatever data can be collected. The BPIC aims at making the research community aware of the existence of these datasets.^7^

The constraints used for analyzing the tools were extracted from the logs provided for the BPIC 2011 [11] and 2012 [12]. The first event log pertains to the treatment of patients diagnosed with cancer in a large Dutch academic hospital. It contains 1143 cases and 150,291 events distributed across 623 activities. Each case in this event log is related to a different patient treated in the hospital. The event log contains domain specific attributes, e.g., *Diagnosis code*, *Treatment code*, *Producer code*, *Diagnosis Treatment Combination ID*, and *Age* in addition to the standard XES attributes for events: *concept:name*, *lifecycle:transition*, *time:timestamp*, and *org:group* [7].

The second event log was recorded for an application process for personal loans or overdrafts in a Dutch financial institute. It merges three intertwined sub-processes. Therefore, in each case, events belonging to different sub-processes can occur. The log contains 262,200 events distributed across 36 activities and includes 13,087 cases. The amount requested by the customer for a loan is indicated in the case attribute *AMOUNT*_*REQ*. In addition, the log contains the standard XES attributes.

#### 5.3.2 Methodology

**Fig. 3:**
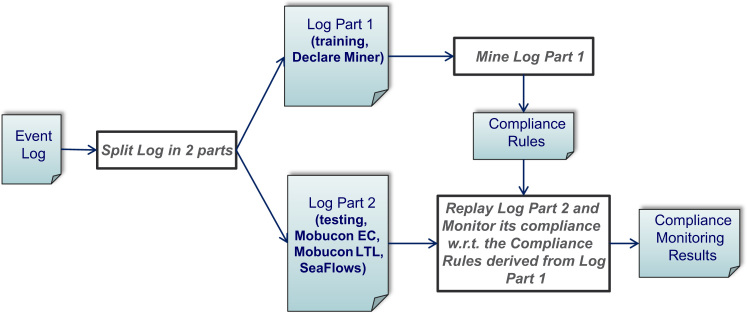
Methodology for tool analysis.

In this section, we illustrate the methodology we have followed to show how the CMFs have been implemented in the selected tools. We started from the data sets described in Section 5.3.1. Fig. 3 illustrates the methodology. We have split the logs in two parts (Log Part 1 and Log Part 2). For the hospital log, in the first part, the first 571 cases are considered. In the second part, the rest of the cases (572 cases) are included. For the financial institute log, in the first part, the first 6543 cases and, in the second part, the rest of the cases (6544 cases) were considered. Then, we have mined Log Part 1 (training log) to extract a set of compliance rules. To do this, we have used the *Declare Miner* component of the process-mining tool ProM [92]. This allows us to automatically discover compliance rules related to control-flow, data and resources based on the mainstream observed behavior, i.e., frequent behavior is converted into a collection of *Declare* constraints. *Declare* is a declarative language based on an extensible set of constraints [87–89,93]. *Declare* supports most of the modeling constructs mentioned in Tables 4–6 and hence a good candidate to evaluate and illustrate the CFMs. The compliance rules have been used as a reference model to monitor a stream of events coming from the replay of Log Part 2 (testing log).

The compliance rules from the BPIC 2011 and 2012 logs are provided next. They are well suitable for a case study as they nicely cover the CMFs. Specifically, they involve quantitative and qualitative time, conditions on both case and activity data as well as on resources. The compliance rules from BPIC 2012 also refer to non-atomic activities and activity lifecycle. Furthermore, some of the presented rules can become conflicting for some specific cases.

*Compliance Rules Discovered from the BPIC 2011 Log*: Through the Declare Maps Miner plug-in of ProM, we have extracted, from the training log derived from the BPIC 2011, the following compliance rules:

R1 If “administratief tarief - eerste pol” occurs in a trace, it is always preceded by “vervolgconsult poliklinisch” and between “administratief tarief - eerste pol” and “vervolgconsult poliklinisch” you cannot find another “administratief tarief - eerste pol”;
R2 If “administratief tarief - eerste pol” or “vervolgconsult poliklinisch” occur in a trace, they always coexist;
R3 If “aanname laboratoriumonderzoek” occurs in a trace, it is always followed eventually by “ordertarief” and vice versa if “ordertarief” occurs, it is always preceded by “aanname laboratoriumonderzoek”;
R4 If “administratief tarief - eerste pol” or “aanname laboratoriumonderzoek” occur in a trace, they always coexist;
R5 If “aanname laboratoriumonderzoek” occurs in a trace, it is never followed by “vervolgconsult poliklinisch”;

Using the Timed Declare Miner plug-in of ProM, we have derived:

R6 If “administratief tarief - eerste pol” occurs in a trace, it may be followed by “beademing - anesthesie - eerste dag” only if from the occurrence of “administratief tarief - eerste pol” at least 30 days and at most 35 days have passed;
R7 If “hemoglobine foto-elektrisch” or “aanname laboratoriumonderzoek” occur in a trace, they always coexist and their time distance is at most 1030 days and at least 1 day;
R8 If “telefonisch consult” or “vervolgconsult poliklinisch” occur in a trace, they always coexist and their time distance is at most 1035 days and at least 1 day;
R9 If “administratief tarief - eerste pol” occurs in a trace, it is always preceded by “vervolgconsult poliklinisch” and “vervolgconsult poliklinisch” occurs at most 1030 days before “administratief tarief - eerste pol”;

Using the Data-Aware Declare Miner plug-in of ProM, we have derived:

R10 If “hemoglobine foto-elektrisch” occurs in a trace and case attribute “Diagnosis Treatment Combination ID” is equal to “495,326”, then “hemoglobine foto-elektrisch” is followed eventually by “ureum”;
R11 If “natrium vlamfotometrisch” occurs in a trace and the condition (over case attributes) “(Age ≥71 && Treatment code ≥ 803 && Diagnosis Treatment Combination ID ≤394,725) ∥ (Treatment code==703 ∥ Treatment code==803))” holds, then “natrium vlamfotometrisch” is not followed eventually by “calcium”;
R12 If “administratief tarief - eerste pol” occurs in a trace and the condition (over case and event attributes) “(Age ≤70 && Producer code==SIOG) ∥ (Diagnosis==Maligne neoplasma cervix uteri && Diagnosis code==106))” holds, then “administratief tarief - eerste pol” is followed eventually by “albumine”;
R13 If “telefonisch consult” occurs in a trace and the condition (over case and event attributes) “(Treatment code==101) && (Producer code==SGAL ∥ Producer code==SGNA)” holds, then “alkalische fosfatase -kinetisch-” does not occur in the same trace.

The same plug-in was used to extract additional resources rules:

R14 If event attribute “Section” is equal to “ Section 4” and event attribute “Specialism code” is equal to “86”, the activity is executed by “org:group==General Lab Clinical Chemistry”;
R15 “bacteriologisch onderzoek met kweek -nie” is always executed by “org:group==Medical Microbiology”;
R16 “cytologisch onderzoek - ectocervix -” and “histologisch onderzoek - biopten nno” are always executed by “org:group==Pathology”;

*Compliance Rules Discovered from the BPIC 2012 Log* Using the training log derived from the BPIC 2012, through the Declare Maps Miner, we have extracted the following compliance rules:

R17 “A_PARTLYSUBMITTED-complete” occurs exactly once in all the traces;
R18 “A_SUBMITTED-complete” occurs exactly once in all the traces;
R19 “A_PARTLYSUBMITTED-complete” is always immediately followed by “A_SUBMITTED-complete” and, vice versa, “A_SUBMITTED-complete” is always immediately preceded by “A_PARTLYSUBMITTED-complete”;
R20 “A_SUBMITTED-complete” and “A_PARTLYSUBMITTED-complete” are always the first two activities in a trace;
R21 The lifecycle “W_Afhandelen leads-schedule”, “W_Afhandelen leads-start”, “W_Afhandelen leads-complete” is always respected;
R22 “W_Completeren aanvraag-schedule” is always followed eventually by “W_Completeren aanvraag-complete” and, vice versa, “W_Completeren aanvraag-complete” is always preceded by “W_Completeren aanvraag-schedule”;
R23 The lifecycle “W_Beoordelen fraude-schedule”, “W_Beoordelen fraude-start”, “W_Beoordelen fraude-complete” is always respected;
R24 “O_SELECTED-complete” is always followed eventually by “O_CREATED-complete”, “O_CREATED-complete” is always followed eventually by “O_SENT-complete” and, vice versa, “O_SENT-complete” is always preceded by “O_CREATED-complete” and “O_CREATED-complete” is always preceded by “O_SELECTED-complete”;
R25 “A_CANCELLED-complete” does not coexist neither with “A_ACTIVATED-complete” nor with “A_REGISTERED-complete” nor with “A_APPROVED-complete” nor with “A_DECLINED-complete”;
R26 “W_Beoordelen fraude-schedule” does not coexist with “W_Wijzigen contractgegevens-schedule”;  item “A_ACCEPTED” and “A_DECLINED” do not coexist.

Using the Timed Declare Miner plug-in of ProM, we have derived:

R27 “A_PARTLYSUBMITTED-complete” occurs at most 22 s after “A_SUBMITTED-complete”;
R28 “W_Completeren aanvraag-complete” occurs at least 22 s and at most 2 days, 18 h, 29 min, and 28 s after “W_Completeren aanvraag-schedule”.

Additional rules about resources were derived using the same plug-in:

R29 “A_SUBMITTED-complete” and “A_PARTLYSUBMITTED-complete” are always performed by the same actor;
R30 “A_ACCEPTED” and “A_FINALIZED” are always performed by the same actor;
R31 “A_SUBMITTED” and “A_FINALIZED” are never performed by the same actor;

#### 5.3.3 Compliance monitoring with MobuconEC

*MobuconEC* is a compliance-monitoring framework based on a reactive version [94] of the Event Calculus [67]. As described in [39,84], the approach has been exploited to formalize the extension of Declare described in [74,84]. This extensions support:

• Metric time constraints, which can be directly formalized (together with qualitative time constraints) using the *explicit approach* to time provided by the Event Calculus (CMF 1: +).
• Data and data-aware conditions, leveraging on the first-order nature of the Event Calculus (CMF 2: +).
• Resources, which are considered as special data (CMF 3: +).
• Non-atomic activities, which can be encoded in the Event Calculus using additional data and dedicated rules, as shown in [39] (CMF 4: +). Notably, the formalization in [39] directly support the possibility of monitoring the lifecycle of activities (CMF 5: +).

In fact, *MobuconEC* is able to express all compliance rules from R1 to R28; the last three rules do not correspond to any data-aware Declare pattern, so they are not directly supported, but could be seamlessly modeled as Event Calculus constraints as well.

**Fig. 4:**
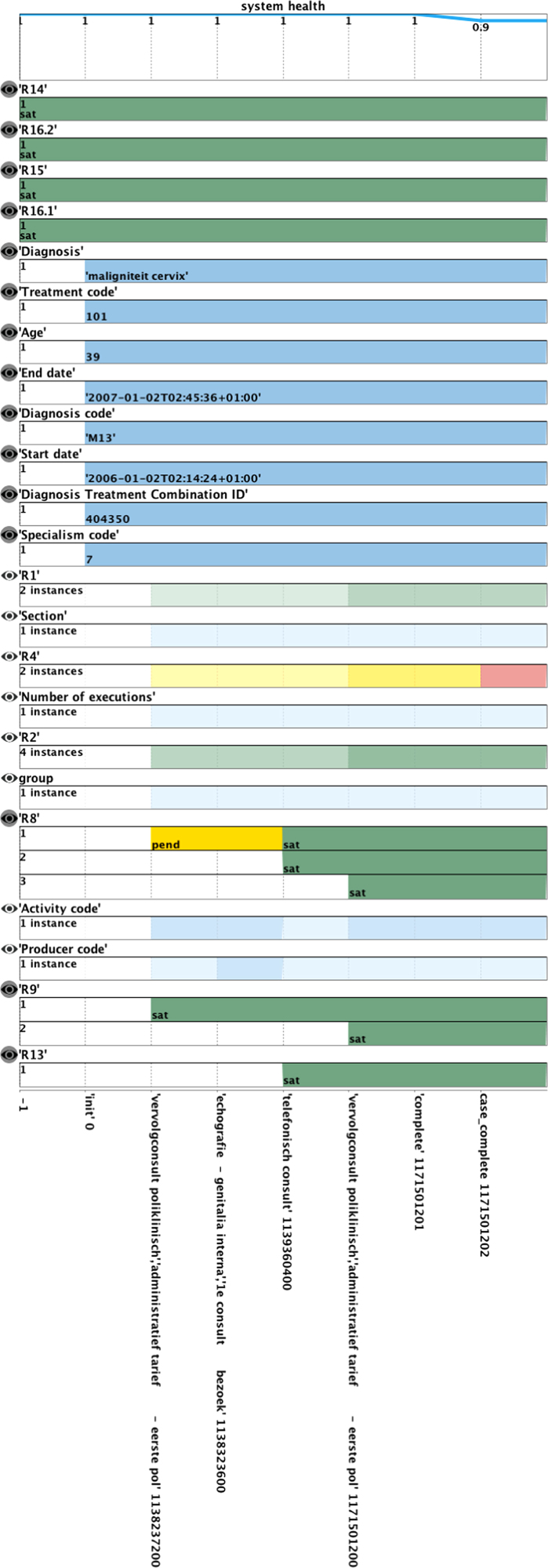
A compliance summary produced by *MobuconEC* when monitoring a trace from the BPI Challenge 2011; some contraints are shown with an expanded view listing the evolution of their instances.

Due to the presence of data and metric time-related conditions, *MobuconEC* does not directly monitor the modeled constraints, but expands them in their different instances, each of which grounds the constraint on a particular context [39]. Consequently, it provides fine-grained compliance checking by analyzing the evolution of each constraint instance (CMF 6: +). In particular, at a given time each active instance could be either satisfied, violated, or pending, the last state meaning that the constraint instance is currently violated, but can still be satisfied by properly continuing the execution of the monitored process (CMF 7: +). In the graphical feedback provided to the end-user, two visualization modes are correspondingly provided for each constraint: a “summary view” in which all instances are packed together, or an “expanded view” in which each single instance is shown separately. These two possibilities are shown in Fig. 4.

This fine-grained analysis of violations at the constraint-instance level constitutes also the basis for quantifying the degree of compliance. For the time being, only simple aggregation metrics are provided to give an indicator about the “global health” of the system (CMF 10: +/−).

The high expressiveness of compliance rules in *MobuconEC* has the main drawback that only reactive management of violations can be tackled: proactive management would require to reason on the possible future continuations of the current, partial trace by considering also data and metric timestamps, which is undecidable (CMF 8: -). Another limitation of the approach is that violations are reported to the user without any additional inference about the corresponding root causes (CMF 9: -).

The latest version of the core *MobuconEC* reasoner with case-data support, implemented in Prolog, is publicly available.^8^ The integration with the operational support backbone of ProM is still ongoing.

#### 5.3.4 Compliance Monitoring with MobuconLTL

*MobuconLTL* is a compliance-monitoring tool implemented as a provider of the operational support in the process-mining tool ProM. It takes as input a reference model expressed in the form of Declare rules. More generally, every business constraint that can be expressed as an LTL formula can be monitored using *MobuconLTL*. A stream of events encoded using XES can be monitored with respect to the given LTL specification.

*MobuconLTL* [37,38] deals with a qualitative notion of time (being based on LTL) but it does not support constraints concerning metric time. In this sense, *MobuconLTL* partially supports CMF 1 (CMF 1: +/−). Therefore, rules expressing qualitative time information (e.g., R1–R5, R17–R26) can be monitored using *MobuconLTL*. On the other hand, rules based on a quantitative notion of time (used to define, e.g., deadlines) cannot be monitored (e.g., R6–R9, R27–R28).

*MobuconLTL* monitors finite-trace LTL constraints through deterministic finite state automata. Therefore, it does not tackle constraints referring to data and resources (ranging over finite state) because of the state space explosion problem (CMF 2: - and CMF 3: -). Therefore, rules involving data and resources cannot be monitored (e.g., R10–R16, R29–R31). With this approach, it is possible to express rules on non-atomic activities (e.g., R17–R26) but it does not fully support the monitoring of activity lifecycle. Indeed, with this approach it is possible to associate an event type to each occurrence of an activity. However, a correlation mechanism to link different events belonging to the lifecycle of the same activity cannot be defined (CMF 5: −). This is, again, related to the impossibility for an automata-based approach of monitoring constraints referring to data. Indeed, the most natural way of implementing such a correlation mechanism would be to connect events with the same value for a certain data (e.g., an activity ID).

**Fig. 5:**
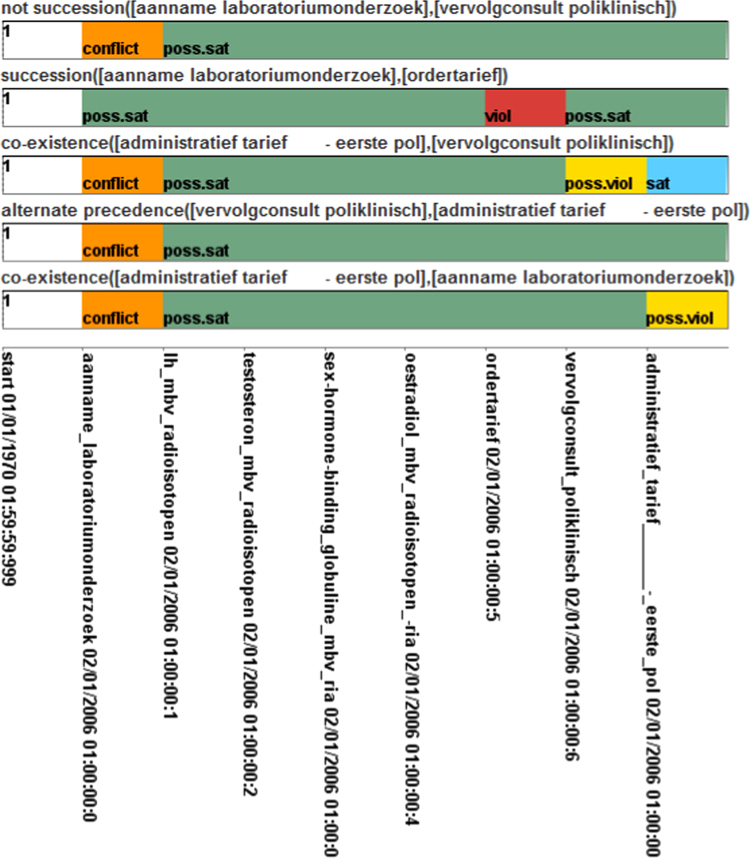
Compliance monitoring with *MobuconLTL*: BPI Challenge 2011.

**Fig. 6:**
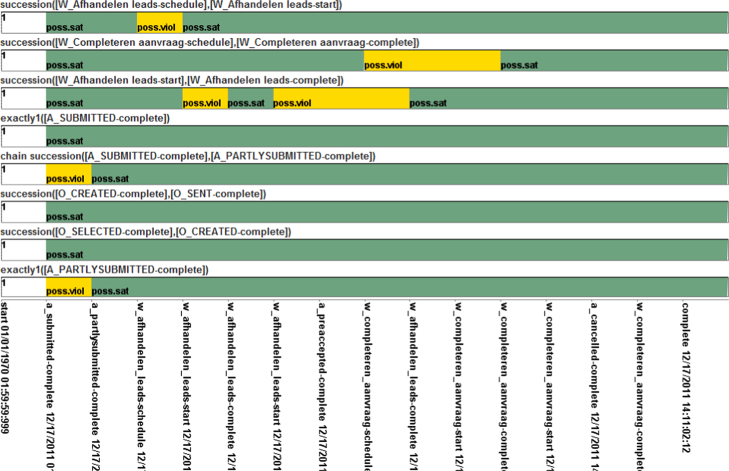
Compliance monitoring with *MobuconLTL*: BPI Challenge 2012.

Fig. 5 represents how rules R1–R5 can be used to monitor with *MobuconLTL* a case replayed from the BPIC 2011 testing log. Fig. 6 shows the monitoring results obtained for a case from the testing log derived from the BPIC 2012 log using the compliance rules R17–R19, R21, R22, and R24. Events are displayed on the horizontal axis. The vertical axis shows the constraints. In particular, each line is labeled with the Declare constraints used to encode the rules mentioned before. Five states are possible for compliance rules: *possibly satisfied, possibly violated, satisfied, violated*, and *conflict*. The first state attests that the monitored case is currently compliant with the rule but can violate the rule in the future. The second state indicates that the compliance rule is currently violated, but it is possible to bring it back to a satisfied state by executing some activity in the future. The third and the fourth states model permanent violations and satisfactions of the rule. In this way, *MobuconLTL* implements reactive monitoring (CMF 7: +). It is possible, at runtime, to detect that a state of affairs is reached such that two or more compliance rules become conflicting (conflict state); the presence of a conflict means that no possible future course of execution exists such that all the involved constraints are satisfied. In this sense, *MobuconLTL* also supports a pro-active management of violations and root cause detection (CMF 8: + and CMF 9: +). For example, in Fig. 5, when *aanname laboratoriumonderzoek* occurs some of the constraints move to a conflict state since some of them require the execution of *vervolgconsult poliklinisch* to be satisfied and for others the execution of this activity is forbidden.

*MobuconLTL* supports continuous monitoring and allows a case to be monitored also after a violation or a conflict has occurred as shown in Fig. 5. One trivial way to implement continuous monitoring is to reset all the automata needed for the monitoring when a violation occurs. Other more sophisticated ways to implement continuous monitoring are presented in [37]. Note that, in the visualization in Fig. 5, different instances of the same rule are condensed in only one line. After every violation and conflict a new instance of the violated rule is started in the same line.

The automata-based approach allows *MobuconLTL* to provide the user with detailed diagnostics about which activities can be executed and which ones are forbidden at any point in time during the process execution. This is possible by evaluating which transitions can be fired from the current state of the automaton and which ones bring the automaton in an inconsistent state. The tool only supports simple metrics for quantifying the degree of compliance of a case [36] (CMF 10: +/−).

#### 5.3.5 Compliance monitoring with SeaFlows

*SeaFlows* is a compliance checking framework that addresses design and runtime checking. It aims at encoding compliance states in an easily interpretable manner to provide advanced compliance diagnosis. The core concepts described in [35,64] were implemented within the prototype named *SeaFlows Toolset*. With *SeaFlows*, compliance rules are modeled as *compliance rule graphs* (CRG). *SeaFlows* enables to monitor a stream of events encoded in a predefined event format. It further enables the import of logs in XES standard format.

Qualitative time constraints are well-supported by *SeaFlows*. In particular, the CRG approach is not restricted to predefined compliance constraint patterns but allows for constraints that are more complex. However, *SeaFlows* does not address quantitative time (CMF 1: +/−). Hence, constraints using metric time (e.g., R6–R9 and R27–R28) are not supported.

*SeaFlows* only partially supports CMF 2 (CMF 2: +/−). This is because it provides only limited support for constraints with non-unary data conditions. Resource-aware compliance rules are only supported if the resource conditions can be expressed via the supported data conditions (CMF 3: +/−). Hence, conditions as required for the resource-related constraints R14–R16 and R29–R30 are not supported. *SeaFlows* supports both atomic as well as non-atomic activities (CMF 4: +) and constraints on the lifecycle of activities like R21–R23 (CMF 5: +).

**Fig. 7:**
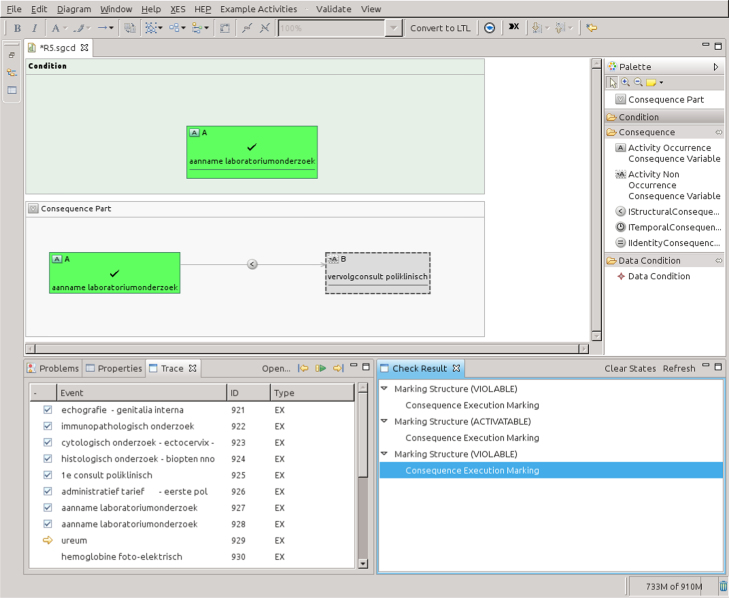
Monitoring with *SeaFlows*: detection of two *violable* activations of a compliance rule.

For this case study, the logs were automatically replayed and checked against all modeled rules. For each violation, a violation file was created. Fig. 7 illustrates how compliance with R5 is monitored by replaying the log of a specific case. As compliance with a CRG is checked by executing it against the event trace using well-defined rules, the compliance state is represented through markings of the CRG. When a violation is observed, it is reflected in the markings of the CRG.

*SeaFlows* supports fine-grained compliance checking by analyzing the individual constraint instances that occur during the process execution (CMF 6: +). In Fig. 7, two instances of R5 were identified by the monitor (state VIOLABLE in the *Check Result* panel in the screenshot). The states of these instances are represented by the marked R5 shown in the main panel in Fig. 7. From this state representation, it is possible to derive information for providing pro-active support in terms of guiding the process execution to avoid violations (CMF 8: +). How this can be done is described in [64]. In the example, activity *vervolgconsult poliklinisch* is associated with an absence node that is next to being executed. Hence, this activity is prohibited and must not be executed as its execution would lead to a violation.

**Fig. 8:**
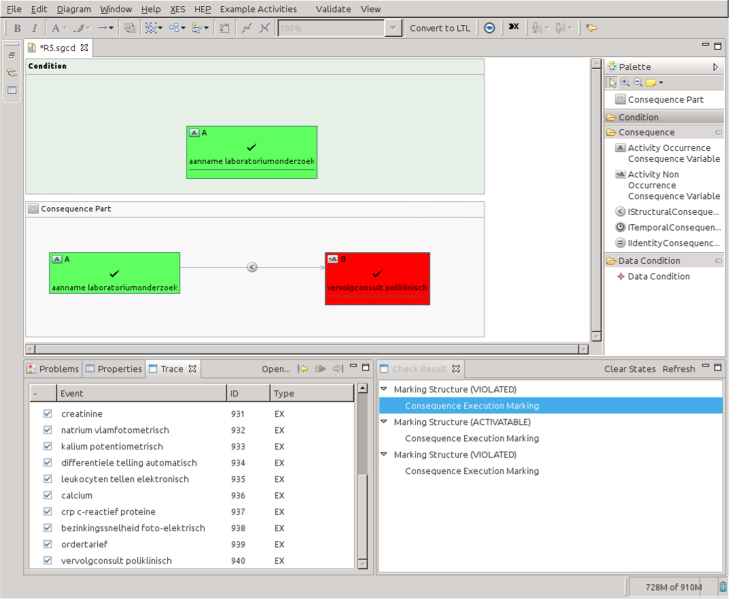
Monitoring with *SeaFlows*: detection of compliance violations.

When the last event of the case, i.e., *vervolgconsult poliklinisch*, is executed, both instances become violated as shown in Fig. 8. The marked CRG serves as basis for deriving explanations for violations (CMF 9: +). In the example, clearly R5 is violated because *vervolgconsult poliklinisch* was executed even though it was prohibited after *aanname laboratoriumonderzoek*. That is the reason why the corresponding node is marked with red color. Once violated, the instance of a compliance rule cannot become satisfied in the further process execution. This is because the violation is already manifest in the log. However, monitoring can still be continued for the compliance rule (i.e., future possible violations of the rule in the process instance can also be detected). Thus, *SeaFlows* supports reactive compliance management (CMF 7: +).

As the compliance monitor of *SeaFlows* was implemented as a proof-of-concept for the concepts described in [64], it does not put emphasis on sophisticated visualization and reporting features for compliance monitoring. Although the compliance states of multiple compliance rule instances can be aggregated to provide an overall compliance level, the framework does not support a more detailed analysis of the individual metrics (CMF 10: +/−). In addition, *SeaFlows* does not detect violations caused by the interplay of two or more constraints. Hence, the conflicts among some of the rules in the case study (cf. Section 5.3.4) remain undetected.

## 6 Conclusion

This paper presents a framework for the systematic comparison of compliance monitoring approaches in the business process management area. The framework consists of *ten Compliance Monitoring Functionalities* (CMFs) and includes requirements for the constraint modeling notation (e.g., supporting time and data), requirements with respect to the execution (e.g., supporting multiple constraint instances), and user requirements (e.g., providing fine-grained feedback). The CMFs are harvested based on a systematic literature review as well as from five case studies from different domains (health care, manufacturing, and maritime safety).

The appropriateness of the CMFF is shown in two ways. First of all, the CMF framework is compared with existing compliance patterns in the business process management area. Secondly, existing compliance monitoring approaches are classified based on their support for the CMFs. The comparison with compliance patterns supports the importance of the four constraint-related CMFs, i.e., those CMFs that relate to language and expressiveness aspects in the constraint specification. The classification of existing approaches pointed out that none of them supports more than seven CMFs and most approaches are not supported by publicly available software tools. Here we have to note that for some approaches several of the CMFs could not be evaluated based on the literature. It seems that several approaches focus on a specific language aspect rather than integral monitoring support. Less attention has been devoted to user requirements, i.e., the provision of fine-grained feedback or even the proactive indication of compliance violations. Nevertheless, it is crystal clear that users play an important role in compliance monitoring, e.g., to interpret deviations.

In order to demonstrate the applicability of our CMF framework, two realistic data sets from the BPIC 2011 and 2012 were applied using three compliance monitoring tools, i.e., *MobuconLTL*, *MobuconEC*, and *SeaFlows*. The data sets consisting of process execution logs from patient treatment and the financial domain were divided into training and testing data sets. Compliance constraints were harvested based on the training set, implemented within the three tools, and monitored over the testing data set. The application of concrete tools to these data sets nicely illustrates how compliance monitoring works in practice and shows what the interaction with users looks like.

Let us now reflect on the four research questions described in the introduction.

• *RQ*1: *How to identify compliance monitoring approaches?* The systematic literature search and complementary case studies helped us to identify typical functionalities required in compliance monitoring.
• *RQ*2: *What are functionalities that are essential for compliance monitoring approaches in business processes?* We identified three essential classes of monitoring functionality, i.e., modeling, execution, and user requirements. However, the CMFF is meant to be extensible, i.e., additional CMFs could become relevant and hence added or existing CMFs might be split or merged. One candidate is CMF 6: supporting multiple-instances constraints, which refers to the multiple instantiation of constraints, but might be also applied to multiple instances of the underlying process (i.e., covering inter-instance constraints as dealt with in, for example, [100]).
• *RQ*3: *How can we demonstrate the appropriateness of the identified compliance monitoring functionalities?* Real event data have been used to illustrate and test the CMFF. However, a real-life case study involving real-time monitoring is still missing. To address this research question better, we need to find a case study where we can embed our monitoring tools in an operational information system.
• *RQ*4: *How can the compliance monitoring functionalities be applied in existing tools?* Three tools were used to demonstrate the practical realization of our CMFF. The choice of the tools was motivated by availability and experience with the tools. A more comprehensive demonstration using additional tools (realizing different approaches) would be useful and balance the viewpoint on the CMFF. For this, the BPI data sets ([11,12], download via DOIs) together with the constraints provided in this paper constitute an available data set that could be used to benchmark compliance monitoring approaches and tools. For a further evaluation of the CMFF, studies with experts from practice are envisaged, e.g., by brainstorming sessions or interviews to assess the appropriateness of the CMFF.

The work can be further extended in several directions, e.g., to cross-organizational or configurable processes. An interesting area of adaptation/extension of the CMF framework here presented naturally arises when there is the need of a comprehensive compliance evaluation not just within, but also across process cases. Traditionally, business processes are monitored in a case-by-case manner. However, compliance rules may span across cases, e.g., because they focus on resources independently from the specific case in which they operate, or because they need to compare and combine data produced inside different cases.

## Figures and Tables

**Table 1:** Literature review: horizontal search.

*Keywords for search*	*#hits*	*#selected*	*Selection criteria*
Process compliance monitoring	13	8	Refers to business processes
Compliance monitoring	813	8	Refers to business processes
Compliance checking	139	11	Refers to business processes and runtime
Compliance audit[ing]	299+137	3+0	Refers to business processes and runtime
Online auditing	37	2	Refers to business processes and compliance
Runtime compliance	3	2	Refers to business processes
Conformance checking	159	5	Refers to business processes, compliance and runtime
Business process compliance	121	28	Refers to runtime, monitoring
Monitoring business constraints	4	3	Refers to processes and compliance
*Results horizontal search:*	1605	70	http://www.wst.univie.ac.at/communities/ComMon/

Added papers		1 [17]	Clear focus on process compliance monitoring
Removed papers		18	Design time, no language requirements, not referring to process constraints

*Results vertical/backward search:*			
Added papers		9 [18–27]	

*Overall:*		60	

**Table 2:** Case studies.

*Domain*	*Project*	*URL*	*Reference*
Health care	“EBMC^2^”	ebmc2.univie.ac.at	[59,60]
Manufacturing	Adventure	www.fp7-adventure.eu	[61]
Higher education	HEP	www.wst.univie.ac.at/communities/hep/	[62]
Maritime safety	Poseidon		[63]
IT project management	SeaFlows	www.seaflows.de	[64]

**Table 3:** CMFF: requirements and CMFs.

**Modeling requirements**	CMF 1: Constraints referring to time
	CMF 2: Constraints referring to data
	CMF 3: Constraints referring to resources

**Execution requirements**	CMF 4: Supporting non-atomic activities
	CMF 5: Supporting activity life cycles
	CMF 6: Supporting multiple instances constraints

**User requirements**	CMF 7: Ability to reactively detect and management
	CMF 8: Ability to pro-actively detect and manage violations
	CMF 9: Ability to explain the root cause of a violation
	CMF 10: Ability to quantify the degree of compliance

**Table 4:** Typical compliance rule patterns dealing with the (co-)occurrence of activities.

**Pattern**	**Description**	**References and Synonyms**
Existence of A	A must be executed at least once	(Global Scope) Existence [43,81,88], Exists [82]
Absence of A	A cannot be executed	(Global Scope) Absence [43,81,88], Absent [82]
Limit A to *N*	A can occur at most *N* times	Absence N [81,88], Limit repetitions [43], Bounded existence [45]
A requires B	If A occurs, then also B must occur	Responded Existence [81,88], CoExists [82], Inclusive [45]
A coexists with B	Either A and B both occur, or none of them does	Coexistence [81,88], CoRequisite [45,82]
A mutex B	A and B cannot both occur	Not Coexistence [81,88], Exclusive [82]
Choose A or B	At least one between A and B must occur	Choice [81,88]
ChooseA xor B	Either A or B must occur, but not both	Exclusive Choice [81,88], MutexChoice [82]

**Table 5:** Typical compliance rule patterns dealing with orderings between activities.

**Pattern**	**Description**	**References and Synonyms**
A followed by B	Whenever A occurs, B must occur afterwards	Response [81,88], After Scope Existence [43], LeadsTo [82]
A precedes B	B can occur only if A occurred before	Precedence [81,88], Before Scope Existence [43], Always precedes [43], Precedes [82]
A blocks B	Whenever A occurs, B cannot occur afterwards	Negation succession [81,88], After Scope Absence [43]
A blocks B until C	Whenever A occurs, C must occur afterwards, and B is forbidden in between	Alternate response (with B=A) [81,88], Between Absence [43]
A immediately followed by B	Whenever A occurs, B must occur next	Chain response [81,88], XLeadsTo [82]

**Table 6:** Typical compliance rule patterns dealing with resources.

**Pattern**	**Description**	**References and Synonyms**
A performed by *R*	A can be performed only by users playing role *R*	PerformedBy [82],
A segregated from B	A and B must be performed by different users	USegregatedFrom [82], 4-Eyes Principle [43]
A fully segregated from B	A and B must be assigned to different roles, and different users must perform them	SegregatedFrom [82]
A bonded with B	A and B must be assigned to the same role, but different users must perform them	RBondedWith [82]
A fully bonded with B	A and B must be assigned to the same role, and the same user must perform them	BondedWith [82]

**Table 7:** Classification of monitoring approaches using the CMFF.

approach	cmf 1	cmf 2	cmf 3	cmf 4	cmf 5	cmf 6	cmf 7	cmf 8	cmf 9	cmf 10
	time	data	resources	non-atomic	lifecycle	multi-instance	reactive mgmt	pro-active mgmt	root cause	compl. degree
Superv. Control Theory [17]	+/−	−	+	+	+	−	−	+	−	−
ECE Rules [31]	+	+/−	+	+	−	−	+	−	+/−	+
BPath (Sebahi) [42]	+	+	+	+	+/−	+	+	−	−	+/−
Gomez et al. [34]	+	−	−	+	n.a.	+/−	+	+	−	−
Giblin et al. [33]	+	n.a.	n.a.	n.a.	n.a.	n.a.	+	n.a.	n.a.	n.a.
Narendra et al. [40]	−	+	+	n.a.	−	+	+	−	−	+
Thullner et al. [41]	+	n.a.	n.a.	n.a.	n.a.	n.a.	+	−	−	n.a.
MONPOLY [26,27]	+	+	+	+/−	+/−	+	+	−	−	−
Halle et al. [24]	+/−	+	+/−	n.a.	n.a.	n.a.	+	n.a.	n.a.	n.a.
Dynamo [21–23]	+	+	+/−	+	n.a.	+	+	−	−	+/−
Namiri et al. [18]	+/−	+	+	+	−	+	+	−	−	−
MobuconEC [39]	+	+	+	+	+	+	+	−	−	+/−
Mobucon LTL [36–38]	+/−	−	−	+	−	−	+	+	+	+/−
SeaFlows [35]	+/−	+/−	+/−	+	+	+	+	+	+	+/−

Caption: + supported, + implementation publicly available, +/−partly supported, −not supported, n.a. cannot be assessed.

## Footnotes

1 Note that other data patterns like scope and block data are too fine-grained in the compliance monitoring setting as it is not known, when monitoring a trace, which were the original notions of blocks/scopes.

2 A precise characterization of which combinations of constructs lead to undecidability of monitoring is outside from the scope of this work. Nevertheless, observe that from the logical point of view, conflict detection requires the ability to check satisfiability of compliance rules in the presence of a partial execution trace. Hence, as soon as data are properly taken into account, that is, compliance rules adopt first-order logic or relational algebra to query the event data, then satisfiability is clearly undecidable even without considering the temporal dimension.

3 For a detailed discussion on the semantical differences between an infinite-trace and a finite-trace settings, see, e.g., [77,78].

4 Note that the patterns *A fully segregated from B*, *A bonded with B*, and *A fully bonded with B* may be enforced at design time by assigning activities to roles accordingly provided that the process-aware information system supports this. However, it is possible to cheat, for example, if different roles are assigned to the same person [52]. Therefore, monitoring these constraints at runtime may still become necessary.

5 This would require to compute satisfiability of MFOTL formulae, which is already undecidable even without considering the temporal dimension.

6 www.processmining.org

7 http://fluxicon.com/blog.

8 https://www.inf.unibz.it/~montali/tools.html.
